# Domestic Summary

**Published:** 1839-12-21

**Authors:** 


### DOMESTIC SUMMARY.

*Black Drop.* By Charles Ellis.—Notwithstanding the discovery of morphia, and its general introduction into use, there are some physicians who prefer the old-fashioned preparation of opium, called black drop.

It becomes important, therefore, to have an article of uniform strength; and that the directions for its preparation should be more in accordance with the modern improvements in pharmacy, than are those which accompany the original recipe. In fact, it could hardly be expected from an adherence to those directions, indefinite and vague as they are, that any certainty in the preparation would be the result. Most of our readers, we presume, are familiar with the old formula, which, with some slight alteration, was published in the first edition of the United States Pharmacopœia. The substitutes which have been offered for black drop, with the advantage of greater certainty in strength, are the acetum opii of the Dublin, and the tinctura opii acetata of the present edition of the United States Pharmacopœia. But in neither of them are the wishes of those who are partial to this preparation met, as they do not produce the rich acetous syrup of opium which was the product of the original prescription.

The following directions, it is believed, will enable the apothecary to preserve the formula in all its essential features, and to prepare black drop without waste of material, and of uniform strength.

℞. Best Turkey opium ℥viij.
White wine vinegar Oiij.
Saffron ℥ss.
Powdered nutmegs ℥iss.
Sugar 1¼ lb.

Rub down the opium with the vinegar, previously made hot; add the saffron, nutmegs, (and if entire conformity with the original be deemed necessary,) ℥j. of yeast; digest them with frequent shaking, for two weeks. Then throw the whole upon a displacement filter, replacing the liquor upon the ingredients until it passes off clear, which, when entirely drained off, measuring about two pints, set aside.

To the ingredients in the funnel, add, a little at a time, Oiss. of vinegar, which, if done with care, will displace the remaining saturated liquor, and thus deprive the ingredients of all their strength, at least, that it is practical or important to obtain.

The second portion of filtered liquor ought to measure considerably less than a pint, and to be equally clear with the first. To it, is to be added one pound, or if a strict adherence to the original formula is preferred, one and a quarter pounds of white sugar. Dissolve with gentle heat, and evaporate slowly to F. Oj. ℥iij., or to a sufficient extent so as to form, when added to the first infusion, exactly F. Oiij. ℥iij. of black drop. Thus prepared, it will contain exactly double the quantity of opium in solution, that is directed in the United States Pharmacopœia for laudanum, admitting the menstruum to be sufficient to take up all the strength of the opium.

The advantages which black drop, properly prepared, possesses over that by the usual method, must be evident. There is absolute certainty, if the opium is good, of having the preparation always the same; there is no waste of material, and the product is a rich, concentrated, aromatic vinegar of opium, as nearly double the strength of laudanum, as the solvent powers of the menstruum will admit of. More than this cannot be anticipated from following the unscientific directions which accompany the original domestic recipe—of boiling all the ingredients up together, and setting aside in the sun for six weeks.

The uncertainty whether the boiling would ever be twice done to the same extent, the difficulty of filtering the preparation after the sugar has been dissolved in it, and the great waste of material, the product being only about half, constitute insuperable objections to the original method, and were the causes which first directed my attention to the subject.—*Journ. of Pharm.*

